# The −174 G/C gene polymorphism in interleukin-6 is associated with an aggressive breast cancer phenotype

**DOI:** 10.1038/sj.bjc.6601545

**Published:** 2004-01-20

**Authors:** B Iacopetta, F Grieu, D Joseph

**Affiliations:** 1 School of Surgery and Pathology, University of Western Australia, Nedlands 6009, Australia; 2 Department of Radiation Oncology, Sir Charles Gairdner Hospital, Nedlands 6009, Australia

**Keywords:** interleukin-6, polymorphism, breast cancer, prognosis

## Abstract

Serum and tissue levels of interleukin-6 (IL-6) have been implicated in the biological phenotype of breast carcinoma. A common G/C polymorphism at position −174 of the *IL-6* promoter can influence the expression level of this gene. We therefore investigated for associations between this polymorphism and various phenotypic features in a series of 256 breast cancers. Individuals who were homozygous for the C allele (*n*=55) were more likely to have higher-grade tumours (*P*=0.039) with ductal histology (*P*=0.030) compared to those harbouring at least one wild-type G allele (*n*=201). Homozygosity for the C allele was also associated with significantly worse overall survival (*P*=0.031). We conclude that the −174 C allele of *IL-6* is associated with a more aggressive breast cancer phenotype.

Interleukin-6 (IL-6) is a pleiotropic growth factor involved in many physiological and pathological processes including carcinogenesis (Ishihara and Hirano, 2002). High serum levels of IL-6 have been associated with advanced stage disease and worse prognosis for several cancer types including ovarian, breast and colorectal (Berek *et al*, 1991; Zhang and Adachi, 1999; Belluco *et al*, 2000; Bachelot *et al*, 2003). In contrast, however, high levels of IL-6 protein and mRNA expression within the breast carcinoma tissue have been linked to better prognosis and to a less malignant phenotype (Basolo *et al*, 1996; Fontanini *et al*, 1999; Karczewska *et al*, 2000).

A common G/C polymorphism located within the *IL-6* promoter at position −174 has been reported to influence IL-6 expression, with the G allele being associated with higher expression levels (Fishman *et al*, 1998; Terry *et al*, 2000; Vickers *et al*, 2002). This polymorphism has been implicated in a number of chronic disease conditions including arthritis, coronary heart disease and diabetes (Fishman *et al*, 1998; Fernandez-Real *et al*, 2000; Yudkin *et al*, 2000). In human cancer, the −174 G/C *IL-6* polymorphism does not appear to be a risk factor for the development of multiple myeloma or melanoma (Zheng *et al*, 2000; Martinez-Escribano *et al*, 2002). Recent data, however, suggest that the C allele is associated with an increased risk of colorectal cancer (Landi *et al*, 2003). Furthermore, in ovarian cancer, the C allele is associated with an earlier stage of disease and with significantly better survival (Hefler *et al*, 2003). In light of the earlier studies linking serum and tissue IL-6 levels to breast cancer outcomes, the aim of the present study was to investigate for possible associations between the −174 G/C *IL-6* polymorphism and phenotypic characteristics of breast cancer.

## MATERIALS AND METHODS

### Breast cancers

Consecutive cases of breast cancer treated surgically between 1992 and 1993 at the Sir Charles Gairdner or Royal Perth Hospitals in Perth, Australia, were selected for study. Genomic DNA was extracted from surgical specimens using standard techniques. The median age of patients at surgery was 59 years (range 18–93 years) and the median follow-up time was 57 months (range 2–96 months). Clinical and pathological features of this tumour series have been described earlier (Soong *et al*, 1997). Approximately 92% of node-positive and 23% of node-negative patients received some form of systemic adjuvant therapy. The majority of patients (>95%) were of European Caucasian descent. Successful genotyping of the −174 G/C *IL-6* polymorphism was achieved for 256 cases. Of these, information on nodal status was unavailable for 61 cases, histological grade for 54 cases, histological type for 23 cases, tumour size for 27 cases, oestrogen and progesterone receptor status for five cases, ploidy for 98 cases, vascular invasion for 45 cases and erbB2 status for 25 cases. An institutional ethics committee approved the study.

### Genotyping for the −174 G/C IL-6 polymorphism

Genotyping for the −174 G/C *IL-6* polymorphism was carried out using a PCR-based fluorescence (F)-SSCP protocol, essentially as described earlier by our laboratory for other single-nucleotide polymorphisms (Powell *et al*, 2002; Grieu *et al*, 2004). HEX-labelled fluorescent primers (Geneworks, Australia) were designed to span the polymorphism and give rise to a PCR product of 165 bp in size. The forward primer sequence was 5′-AGGAAGAGTGGTTCTGCTTC-3′ and the reverse primer sequence 5′-CTTTGTTGGAGGGTGAGGGTG-3′. PCR was carried out in a volume of 15 *μ*l comprising a mix of 1 × reaction buffer, 0.2 mM dioxynucleotide triphosphates, 2.5 mM MgCl_2_, 0.4 *μ*M of each primer and 0.3 U *Taq* polymerase (Qiagen, Australia). Samples were heated to 94°C before addition of DNA template 200 (200 ng). PCR comprised 10 min of denaturation at 94°C followed by 32 cycles of 45 s at 94°C, 45 s at the 64°C annealing temperature and 45 s at 72°C. Final extension was at 72°C for 5 min.

For F-SSCP, 2 *μ*l of PCR product was mixed with 4 *μ*l of deionised formamide-loading buffer and denatured at 94°C for 3 min. A volume of 1 *μ*l of this mix was then loaded onto a nondenaturing 10% polyacrylamide gel and run on the Gel-Scan 2000 DNA fragment analyzer, according to the manufacturer's instructions (Corbett Research, Australia). The sample was pulse loaded for 20 s at 1200 V, the wells rinsed and the gel run for 90 min at 1200 V in 0.8 × TBE buffer at a constant temperature of 22°C. Sequencing of four DNA samples displaying homozygous banding patterns on F-SSCP gels was carried out in order to identify the three possible −174G/C *IL-6* genotypes.

### Statistical analysis

The *χ*^2^ test (Pearson statistic) was used to determine associations between the −174 G/C *IL-6* polymorphism and various clinical and pathological features of the breast tumours. Kaplan–Meier analysis was used to assess the cumulative survival probabilities and differences were evaluated using the log-rank test. Cox regression was used in univariate survival analysis of various established prognostic factors and for the −174G/C *IL-6* genotype. All *P*-values are derived from two-tailed statistical tests. Analyses were carried out using the SPSS statistical software package (Chicago, IL, USA).

## RESULTS

**Figure 1:**
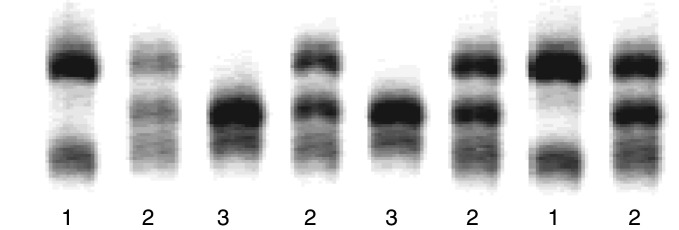
F-SSCP genotyping for the −174 G/C *IL-6* polymorphism in breast cancer patients. The three different genotypes are indicated by the banding patterns observed in the gels: (1) CC homozygote; (2) CG heterozygote; (3) GG homozygote. Not all bands seen by F-SSCP are shown.

Three distinct banding patterns could be seen for the −174 G/C *IL-6* polymorphism using the F-SSCP genotyping method (Figure 1

). DNA sequencing identified these as CC homozygotes (pattern 1), GG homozygotes (pattern 3) and the GC heterozygote (pattern 2). Frequencies for the C and G alleles were 0.43 and 0.57, respectively, and were in Hardy–Weinberg equilibrium. The C allele frequency has previously been reported as 0.41 in a large (*n*=1109) community-based Australian study (Chapman *et al*, 2003), 0.43 in a UK study of 588 individuals (Vickers *et al*, 2002), 0.40 in another UK study of 383 healthy individuals (Fishman *et al*, 1998) and 0.43 in a study of 121 ovarian cancer patients from Austria (Hefler *et al*, 2003).

Tumours showing nonductal histology or well-differentiated morphology were significantly under-represented in patients who were homozygous for the C allele (Table 1

**Table 1:** The −174 G/C *IL-6* polymorphism and breast cancer phenotype

**Feature (*n*)**	**CC (%)**	**CG (%)**	**GG (%)**	***P*^a^**	***P*^b^**
Total (256)	55 (21)	112 (44)	89 (35)		
Age ⩽57 years (117)	23 (20)	48 (41)	46 (39)		
Age >57 years (139)	32 (23)	64 (46)	43 (31)	NS	NS
Node negative (102)	20 (20)	52 (51)	30 (29)		
Node positive (93)	20 (22)	38 (41)	35 (38)	NS	NS
Well differentiated (29)	2 (7)	13 (45)	14 (48)		
Mod./poorly differentiated (173)	39 (23)	74 (43)	60 (35)	0.037	0.039
Non-ductal histology (25)	1 (4)	14 (56)	10 (40)		
Ductal histology (208)	47 (23)	89 (43)	72 (35)	0.045	0.030
Tumour size ⩽20 mm (121)	20 (17)	55 (45)	46 (38)		
Tumour size >20 mm (108)	29 (27)	45 (42)	34 (31)	0.066	0.057
High oestrogen receptor (170)	32 (19)	74 (43)	64 (38)		
Low oestrogen receptor (80)	23 (29)	35 (44)	22 (27)	0.044	0.077
High progesterone receptor (158)	34 (22)	69 (44)	55 (34)		
Low progesterone receptor (92)	21 (23)	40 (43)	31 (34)	NS	NS
Diploid (62)	12 (19)	26 (42)	24 (39)		
Aneuploid (96)	28 (29)	41 (43)	27 (28)	0.098	0.17
No vascular invasion (144)	32 (22)	59 (41)	53 (37)		
Vascular invasion (67)	11 (17)	31 (46)	25 (37)	NS	NS
ErbB2 normal (192)	36 (19)	91 (47)	65 (34)		
ErbB2 amplified (39)	12 (31)	13 (33)	14 (36)	NS	0.092
Normal p53 (214)	44 (21)	92 (43)	78 (36)		
Mutant p53 (42)	11 (26)	20 (48)	11 (26)	NS	NS

a *P* CC *vs* GG.

b *P* CC *vs* CG/GG.

). Only one out of 25 (4%) tumours with nonductal histology were found in CC homozygous patients compared to 47 out of 208 (23%) tumours with ductal histology (*P*=0.03). CC homozygous patients also showed trends for association with larger tumour size, low oestrogen receptor content, aneuploidy and amplified ErbB2 status, all of which are known features of poor prognosis. No associations were seen, however, with two other features of poor prognosis: positive nodal status and the presence of mutant p53.

**Figure 2:**
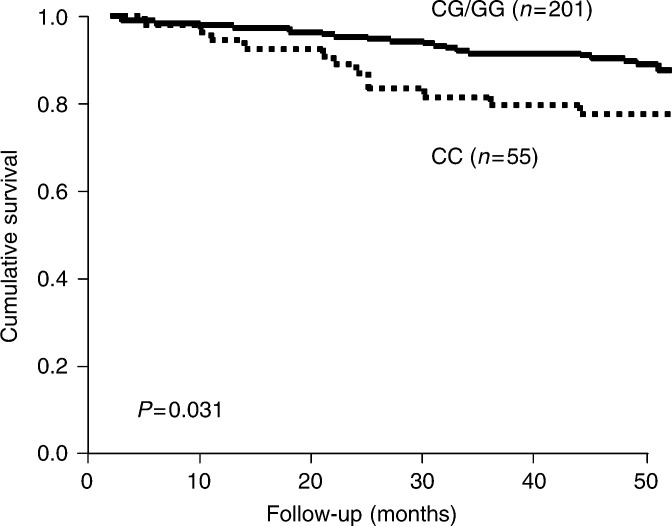
Kaplan–Meier analysis of the overall survival of breast cancer patients grouped according to their genotype for the −174 G/C *IL-6* polymorphism.

Kaplan–Meier survival analysis revealed the *IL-6* −174 CC genotype was associated with significantly worse overall survival compared to the GG or GC genotypes (*P*=0.031, Figure 2

). The increased relative risk of death associated with homozygosity for the C allele was shown in univariate survival analysis to be 1.99 (95% confidence interval: 1.05–3.77; Table 2

**Table 2:** Univariate survival analysis of established prognostic features and of the −174G/C IL-6 genotype

**Feature**	**Relative risk (95% CI)**	***P***
−174G/C *IL-6* genotype^1^	1.99 (1.05–3.77)	0.034
Nodal involvement	2.44 (1.66–3.57)	<0.001
Histological grade	3.28 (1.84–5.85)	<0.001
Tumour size	4.44 (2.19–9.00)	<0.001
Tumour type	1.24 (0.62–2.51)	NS
Oestrogen receptor	1.05 (0.82–1.33)	NS
Progesterone receptor	1.03 (0.82–1.30)	NS
Ploidy	0.99 (0.58–1.70)	NS
ErbB2 amplification	2.05 (1.04–4.03)	0.037

CC *vs* CG/GG genotypes. For all other factors, the groups compared are the same as those shown in Table 1.

). The established features of nodal involvement, poor histological grade, large tumour size and ErbB2 amplification were all found to be associated with poor outcome in this tumour series (Table 2). Interestingly, the CC genotype was over-represented in patients whose tumours showed the three latter characteristics (Table 1). The CC genotype was not an independent factor for poor survival, however, in a multivariate model that included nodal status, histological grade, tumour size and ErbB2 amplification.

## DISCUSSION

Epithelial cells of the normal mammary gland constitutively produce several cytokines including IL-6 (Basolo *et al*, 1993). These have been proposed to play a role in the growth and differentiation of mammary epithelial tissue. The levels of IL-6 mRNA and protein are strongly reduced in invasive ductal carcinomas, suggesting an inverse relationship between tumour aggressiveness and the expression of this cytokine (Basolo *et al*, 1996; Fontanini *et al*, 1999; Karczewska *et al*, 2000). Since the common −174 G/C polymorphism has been linked to reduced production of IL-6 (Fishman *et al*, 1998), we hypothesised that this genetic variant may be associated with a more aggressive breast cancer phenotype.

We observed that homozygosity for the −174 C allele was significantly associated with poor histological grade and with ductal histology, and showed trends for association with larger tumour size and low oestrogen receptor content (Table 1). In keeping with this, CC homozygous patients also showed worse overall survival compared to the GC/GG patient group (Figure 2), although this was not independent of other established prognostic factors. Our results indirectly support the earlier observations of Basolo and co-workers, who showed that a low tumour level of IL-6 correlated with a more aggressive phenotype (Basolo *et al*, 1996; Fontanini *et al*, 1999). The C allele of the −174 G/C polymorphism has been shown to have lower *IL-6* transcription rates and to be associated with lower expression levels compared to the G allele (Fishman *et al*, 1998; Terry *et al*, 2000; Vickers *et al*, 2002). This might explain our observation of an association between the CC genotype and a more aggressive breast cancer phenotype. Confirmation of this will require direct comparison between the −174 G/C genotype and tumour IL-6 expression levels.

At least two other studies have reported an association between low tissue expression of IL-6 and aggressive tumour behaviour (Gandour-Edwards *et al*, 1995; Basolo *et al*, 1998). Apart from the current study, however, there has been only one report of an association between the *IL-6* −174 G/C polymorphism and tumour phenotype (Hefler *et al*, 2003). In contrast to the present results on breast cancer, these workers found that the C allele was associated with low stage and good prognosis in ovarian cancer patients. A possible explanation may be that IL-6 has different roles in carcinogenesis according to the tumour type.

Elevated serum levels of IL-6 have been associated with increased tumour burden and more advanced disease in several cancer types including colorectal (Belluco *et al*, 2000), ovarian (Berek *et al*, 1991) and breast (Zhang and Adachi, 1999; Bachelot *et al*, 2003). In a recent study of colorectal cancer, high serum IL-6 levels were linked to the −174 GG genotype, but only in patients with advanced disease (Belluco *et al*, 2003). It is therefore unclear whether elevated serum levels of IL-6 are a consequence of or a contributory cause to advanced tumour stage. Further prospective studies will be required to elucidate the relationships between the −174 G/C polymorphism, serum IL-6 levels, normal and tumour tissue IL-6 levels, and the biological phenotype for different cancers. It will also be interesting to determine whether the −174 C allele is a risk factor for breast cancer, as was shown recently for colorectal cancer (Landi *et al*, 2003).

We have previously shown significant associations in this breast tumour cohort between polymorphisms in the *p53*, *p21* and luteinising hormone receptor genes and various phenotypic features including histological grade, steroid receptor level and tumour size (Powell *et al*, 2002,^2003^). The present results provide further evidence that genetic variants associated with known functional alterations can influence tumour phenotype.
